# Aggregation and Oligomerization Characterization of ß-Lactoglobulin Protein Using a Solid-State Nanopore Sensor

**DOI:** 10.3390/s24010081

**Published:** 2023-12-22

**Authors:** Mitu C. Acharjee, Brad Ledden, Brian Thomas, Xianglan He, Troy Messina, Jason Giurleo, David Talaga, Jiali Li

**Affiliations:** 1 Material Science and Engineering, University of Arkansas, Fayetteville, AR 72701, USA; 2 Department of Physics, University of Arkansas, Fayetteville, AR 72701, USA; 3 Department of Chemistry and Chemical Biology, Rutgers, The State University of New Jersey, Piscataway, NJ 08854, USA; xianglhe@eden.rutgers.edu (X.H.); jason.giurleo@regeneron.com (J.G.);; 4 Department of Physics, Berea College, Berea, KY 40404, USA; 5 Regeneron Pharmaceuticals, Tarrytown, NY 10591, USA; 6 Department of Chemistry, Sokol Institute, Montclair State University, Montclair, NJ 07043, USA

**Keywords:** protein aggregation, solid-state nanopore, ß-lactoglobulin, protein oligomerization, protein volume

## Abstract

Protein aggregation is linked to many chronic and devastating neurodegenerative human diseases and is strongly associated with aging. This work demonstrates that protein aggregation and oligomerization can be evaluated by a solid-state nanopore method at the single molecule level. A silicon nitride nanopore sensor was used to characterize both the amyloidogenic and native-state oligomerization of a model protein ß-lactoglobulin variant A (βLGa). The findings from the nanopore measurements are validated against atomic force microscopy (AFM) and dynamic light scattering (DLS) data, comparing βLGa aggregation from the same samples at various stages. By calibrating with linear and circular dsDNA, this study estimates the amyloid fibrils’ length and diameter, the quantity of the βLGa aggregates, and their distribution. The nanopore results align with the DLS and AFM data and offer additional insight at the level of individual protein molecular assemblies. As a further demonstration of the nanopore technique, βLGa self-association and aggregation at pH 4.6 as a function of temperature were measured at high (2 M KCl) and low (0.1 M KCl) ionic strength. This research highlights the advantages and limitations of using solid-state nanopore methods for analyzing protein aggregation.

## 1. Introduction

Protein aggregation is linked to many chronic and devastating neurodegenerative human diseases [1,2] and is strongly associated with aging [3]. There are over 20 diseases associated with pathological protein aggregation including Alzheimer’s, Parkinson’s, and type II diabetes [4,5,6,7]. Protein aggregation is also a major concern for protein-based biopharmaceuticals because it can potentially affect drug activity and trigger allergic responses in patients [8]. It is challenging to characterize aggregated proteins due to their heterostructure forms [9] such as dimer, tetramer, hexamer, octamer, filaments, etc. Protein aggregation can be evaluated with ensemble methods such as analytical ultracentrifugation, field flow fractionation [8,10], size-exclusion chromatography, gel electrophoresis, and dynamic light scattering [11,12]. These ensemble techniques provide average protein aggregation states but no information on each protein molecule. As such, they rely on the deconvolution of signals to resolve the aggregate size. Protein aggregation has also been measured by single molecule methods such as atomic force microscopy (AFM) [11]. However, AFM measurements require a protein sample to be on a solid surface, and this requirement could influence the protein aggregation state.

This study employs a silicon nitride nanopore-based sensor for characterizing protein aggregation under varied solution conditions. Solid-state nanopore based devices [13,14,15,16], previously used for analyzing single molecules of DNA [17,18,19,20,21,22,23,24,25,26,27], RNA [28,29,30,31,32,33,34], proteins [35,36,37,38,39,40,41,42,43,44,45,46,47,48,49,50,51,52,53,54,55,56], protein–DNA complexes [57,58,59,60,61,62,63,64,65,66,67,68,69], as well as protein oligomerization [69] and interaction with other analytes [70,71]. For this study, a chief advantage of the solid-state nanopore method is that the measurement of protein aggregation can be performed under solution conditions very close to the native environment, while varying temperature, pH, and salt concentration at the single molecule level. This approach aims to provide a method for more the accurate and detailed understanding of protein aggregation.

**Figure 1:**
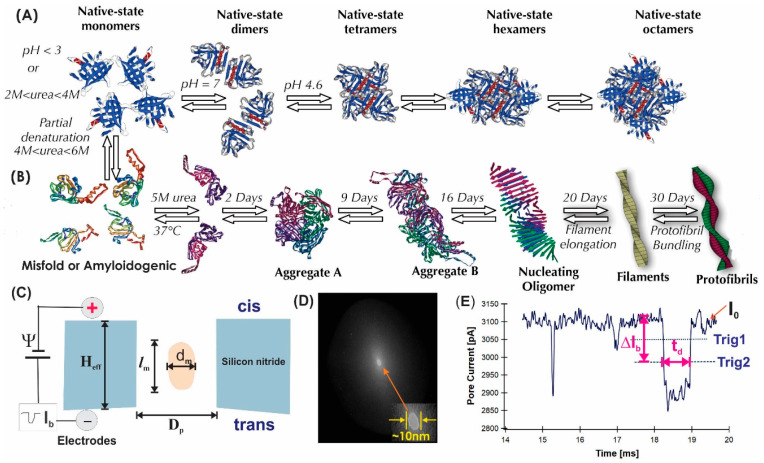
(**A**) ß-lactoglobulin native-state oligomerization. (**B**) Amyloidogenic aggregation of 1 mg/mL (55 µM) ßLGa incubated in 5 M Urea, 10 mM phosphate buffer at pH 7.0 and 37 °C. (**C**) An illustrated nanopore experimental setup for characterization of proteins in globular shape. (**D**) A TEM image of a ~10 nm diameter silicon nitride nanopore used in this work for Day-1 and Day-10 samples. (**E**) An example event recorded from a Day-10 sample of βLGa in 2 M KCl and 5 M Urea.

In this study, a cow’s milk protein, ß-lactoglobulin variant A (βLGa MW = 18.3 kDa/monomer), serves as our model protein. There are many possible species of βLGa amyloid particles present physiologically. The species that are relevant to this work are shown in Figure 1A,B. The βLGa protein aggregation species can be classified into two categories: one is the native-state oligomers (top panel), the other is the amyloidogenic aggregates (bottom panel). When βLGa is at a native state (no denaturing agent present in solution), it can present as monomers, dimers, tetramers, hexamers, and octamers (Figure 1A). When βLGa is incubated in 5 M urea (partially denatured condition) at 37 °C, depending on the incubation time (Figure 1B), it can be in the form of a native-state monomer, partially denatured monomer, completely unfolded monomer, forming dimers through disulfide bonding, and further forming tetramers (Aggregate A), octamers (aggregate B), amyloid filaments (a species of amyloid with diameter of 3–6 nm and length <100 nm), and amyloid fibrils ~10 nm in diameter and >100 nm in length. Interest in βLGa aggregation and its amyloid growth has been driven by basic science, by the importance of βLGa to the dairy and food processing industries, and by finding the mechanism of protein aggregation related to diseases. Detecting these βLGa aggregation and oligomerization species under different solution conditions will allow us to evaluate the advantages and limitations of the solid-state nanopore method. 

The aim of this study was to demonstrate that a solid-state nanopore device can be used to characterize the species (Figure 1A,B) involved in βLGa protein native-state oligomerization and amyloid formation at the single molecule level under near-native conditions. Linear and circular dsDNA as reference molecules of known dimensions were also measured in the same nanopores used to estimate the βLGa aggregation states.

To validate the nanopore approach, we used a combination of atomic force microscopy (AFM) and dynamic light scattering (DLS) techniques in parallel to measure the βLGa protein samples. The strategy here was to allow for the overlapping capability of AFM and DLS to connect all the ranges of the particle sizes. AFM is less-suited for small, partially denatured proteins, but resolves medium-to-large aggregates and fibrils quite well. Conversely, DLS provides good information about small-to-medium-sized aggregates, but those signals become lost once large particles such as protofibrils are present in the sample. 

## 2. Materials and Methods

### 2.1. The Principle of Detecting Protein Aggregation by Nanopore Technolgy

The principle of nanopore characterization of protein aggregation is illustrated in Figure 1C. The main component of a solid-state nanopore sensing system is a single nanometer pore fabricated in silicon nitride membrane that separates two salt solution-filled chambers, the only connection of which was via the electrolyte solution inside the nanopore. A TEM image of a ~10 nm diameter silicon nitride nanopore is shown in Figure 1D. Briefly, when a voltage Ψ is applied through a pair of AgCl electrodes across the membrane with a nanopore submerged in a salt solution, a stable open pore current, $I_{0}=\Psi /R_{pore}$, can be established by the flowing of ions and measured ($R_{pore}$ is the pore resistance). When charged protein molecules are present in the *cis* chamber, the electric field of right polarity near the nanopore can capture the protein molecules and drive them through the pore to the *trans* side, one at a time, if the right size of nanopore is selected. A protein molecule translocating through a nanopore partially blocks the flow of ions that would increase the pore resistance (decrease the pore current) and produce a current blockage pulse or event, as shown in Figure 1E.

Previous studies with particles of various shape and size that translocate through various sizes of pores [72,73,74,75] have shown that the instantaneous current blockage amplitude ∆*I*_b_(t) is approximately proportional to the instantaneous excluded volume Λ(t) of the passing DNA or protein molecule, as well as the applied voltage Ψ, as described in Equation (1) [18,76]

(1) $$∆I_{b}≃I_{0}\frac{{\gamma \Lambda }_{Pr}}{V_{po}}=(\frac{\gamma \sigma \Lambda_{Pr}}{{H_{eff}}^{2}})\Psi$$

Here, by Ohm’s law, $I_{0}=\Psi /R_{pore}=(\sigma A_{p0}/H_{eff})\Psi$, *σ* is the solution conductivity (see Appendix A for all *σ* used in this work), *γ* is a shape factor of a protein molecule [45,46], *V*_po_ = *A*_po_ × *H*_eff_ is the nanopore effective volume of pore area *A*_po_ (determined by TEM image) and an effective thickness, *H*_eff_. The *H*_eff_ can be determined by the slope $\left(\sigma A_{p0}/H_{eff}\right)$ of $I_{0}$ vs. Ψ curve (see Appendix B Table A1 for all *H*_eff_ determined in this work) and is usually larger than its physical thickness because of the access resistance of a nanopore. By using a standard molecule that has a known diameter, dsDNA for example, we can calibrate the effective thickness, *H*_eff_ (or *V*_po_), of the nanopore under the experimental solution conditions. By measuring the relative current drop, $∆I_{b}/I_{0}$, the product of the volume and shape factor, ${\gamma \Lambda }_{Pr},$ of a translocating protein can be estimated. For example, a tetrameric aggregate will have approximately 4 times larger relative current drop than a monomer, βLGa, $∆I_{b}/I_{0}$ (tetramer) ≈ 4$∆I_{b}/I_{0}$ (monomer).

In this study, we focus on measuring the relative current drop amplitudes, ∆*I*_b_/*I*_0_, to probe the βLGa protein native-state oligomerization and amyloid assembly. We first present nanopore characterization of βLGa protein aggregation at different stages under amyloid formation conditions (in 5 M urea at 37 °C, Figure 1B). DLS was used to measure and verify the number of aggregates and AFM was used to measure and calibrate the dimensions of the βLGa aggregates from the same stages. Furthermore, we demonstrate the characterization of βLGa protein native-state oligomerization (Figure 1, top panel) as a function of solution temperature at biological salt concentration (0.1 M KCl) and high salt concentrations (2 M KCl).

### 2.2. Materials and Sample Preparation

#### 2.2.1. Nanopore Experiment

The fabrication and characterization of silicon nitride nanopores have been described in detail in our previous work [20,76,77]. The thickness of the silicon nitride nanopores fabricated was estimated to be 10 to 30 nm, depending on the ion species used [77,78,79]. The diameters of the nanopores used in this work were from 8 to 16 nm, selected based on the size of the protein aggregates to be measured. The parameters of the 3 nanopores used in this report are listed in the Supporting Information (S-I-Table S1). A pair of Ag/AgCl electrodes is embedded in each PDMS (polydimethylsiloxane) chamber [20].

Current blockage event traces were recorded with an Axopatch 200B (Molecular Devices, San Jose, CA, USA) integrated system in event-driven mode with its low-pass Bessel filter set at 10 kHz. The recorded events were sorted, and the current drop amplitudes (∆*I*_b_) and dwell times (*t*_d_) were extracted using a home-made MATLAB program. The events analyzed here were those that had the current dropping below both Trig 1 and Trig 2, then came back above Trig 1 (Figure 1E) with *t*_d_ > 35 µs. Trig 1 was set just below the baseline noise level and Trig 2 was set at approximately half the average peak height where *t*_d_ was measured. For those events with *t*_d_ < 35 µs, no meaningful parameters can be extracted due to the 10 kHz filter. A total of 200 data points before and after each event were also recorded to determine the open pore current for the event. When using a low-pass Bessel filter set to 10 kHz, pulse widths shorter than 100 µs maintain accurate time durations (*t*_d_, fwhm), but the pulse heights are attenuated. Pulse height attenuation for short *t*_d_ (<100 μs) was corrected, as described in our previous publication [23]. The applied voltage was set at Ψ = 120 mV for all nanopore measurements, unless otherwise mentioned.

#### 2.2.2. Data Analysis

As illustrated in Figure 1E, the current blockage amplitude, ∆*I*_b_, was calculated by averaging the current during the dwell time, *t*_d_, corresponding to the data points below the Trig 2. Each event is represented by one point in plots of ∆*I*_b_ vs. *t*_d_ in this paper. The peak values of ∆*I*_b_ and *t*_d_ were obtained from fitting their histograms to Gaussians (or multi exponentials for *t*_d_). Their errors were half the width of the fits.

#### 2.2.3. AFM and DLS Measurements

The sample preparation, as well as AFM and DLS measurements have been described in detail in Giurleo et al. [11]. Below, we summarize them briefly. 

*AFM*. The βLGa samples were imaged on mica surfaces by a MultiMode Scanning Probe Microscope (Digital Instruments, New York, NY, USA) with a TESP tip in tapping mode. To obtain better adhesion of protein aggregates to the mica surface, chemical surface modification with (3-aminopropyl) triethoxysilane was implemented, as described in Giurleo et al. [11]. 

*DLS*. Fluctuations of scattered light intensity were measured using a homodyne technique. Round borosilicate glass cuvettes (Kimble Glass, Düsseldorf, Germany) were used for all DLS measurements. For the DLS study (details in S-II), 250 μL of incubated sample was placed in a clean dry cuvette. Twenty correlation functions were measured sequentially for 30 s apiece for the incubated sample. The cuvette chamber was held at a constant temperature of 37 °C. 

#### 2.2.4. Chemicals

ßLGa, urea, KCl, and dibasic and monobasic sodium phosphate were purchased from sigma-Aldrich. βLGa at a concentration of 1 mg/mL (~55 µM) was incubated in 5 M urea, 10 mM phosphate buffer at pH 7.0 and 37 °C. The Day-1, Day-10, and Day-29 samples were measured on day 1, days 10–11, and days 29–31 of incubation, respectively. The reference DNA molecules, 2.7 kbp linear dsDNA, and 5.4 kbp circular dsDNA were purchased from New England Biolabs.

## 3. Results and Discussion

### 3.1. LGa Aggregation and Amyloid Formation

#### 3.1.1. Nanopore Measurements of ßLGa Incubated on Day 1 and Day 10

In a solution of 2 M KCl with 5 M urea and 10 mM phosphate buffer at pH 7.0 and 20 °C, at applied voltage Ψ= 120 mV, the ~10 nm nanopore shown in Figure 1D had an open pore current $I_{0}=3.1\;nA$. The incubated βLGa protein of Day-10 and freshly prepared Day-1 samples were diluted 550 times to a final concentration of ~100 nM. The Day-10 sample was added to the cis chamber and the current blockage events were observed in a few minutes. After about ten thousand events were recorded, the cis chamber solution was flushed out several times until no events were observed, and the freshly made Day-1 sample was added to the cis chamber and measured. 

**Figure 2:**
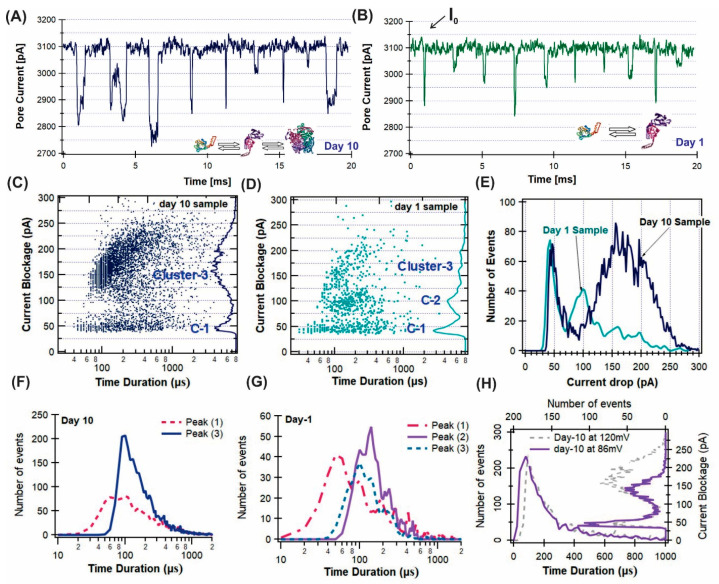
Ionic current traces recorded for Day-10 (**A**) and Day-1 (**B**) samples measured in a ~10 nm pore (TEM image is shown in 1D) in 2 M KCl, 10 mM Phosphate, 5 M Urea, at pH 7. The inserts are possible forms of ßLGa species. The open pore current was *I*_0_~3.1 nA at 120 mV. Event distributions displayed as current blockage amplitude, Δ*I*_b_, versus time duration, *t*_d_, for the samples from Day 10 (**C**) and Day 1 (**D**). (**E**) Histograms of Δ*I*_b_ for the Day-10 and Day-1 samples. Time duration histograms of *t*_d_ for the two clusters of events for the Day-10 sample (**F**) and three clusters of events for the Day-1 sample (**G**). (**H**) Comparison of Δ*I*_b_ and *t*_d_ histograms at Ψ = 120 mV and 86 mV.

The traces from the Day-10 sample (Figure 2A) show more current drop events with a larger current drop (∆*I*_b_) and longer durations (*t*_d_) compared to those from the Day-1 sample (Figure 2B). Both the Day-10 and Day-1 samples show variations in the current drop amplitudes, ∆*I*_b_, and time durations, *t*_d_, suggesting that several species of βLGa protein existed in the solution. For the Day-10 sample, the event distribution plot of ∆*I*_b_ versus *t*_d_ in Figure 2C shows two main clusters of events, labeled as cluster-1 and cluster-3. The histogram of ∆*I*_b_ on the right axis (Figure 2C) shows a broad peak for the cluster-3 events, indicating a wide range of βLGa aggregate sizes. For the Day-1 sample, the event distribution plot shows three clusters of events (Figure 2D). We selected each cluster of events and fit their ∆*I*_b_ (Figure 2E) and *t*_d_ (Figure 2F,G) histograms with Gaussians. The fitting peak values and their fitting errors are listed in Table 1. 

Through examining the number of events vs. ∆I_b_ distribution for the Day-1 and Day-10 samples (Figure 2E), the ratio of cluster-3/cluster-1 was found to be approximately 12 times larger for the Day-10 than the Day-1 sample, indicating the βLGa species in cluster-3 had grown significantly during the 10-Day incubation. Further analysis shows that the ratio of the current blockage amplitude peak values of cluster-3 to cluster-1 were ∆*I*_b,3_/∆*I*_b,1_~3.6 for both the Day-10 and Day-1 samples. Therefore, we conclude that the cluster-3 events represented the βLGa protein aggregates that had grown in the incubated sample. 

Assuming the shape factors, γ, in Equation (1) were the same, the excluded volume of cluster-3 events was about four times larger than the cluster-1 events, or $\Lambda_{Pr}$^3^/$\Lambda_{Pr}$^1^~4, so the aggregation number was most likely to be n = 4 (Aggregate A in Figure 1). The cluster-2 events in the Day-1 sample, ∆*I_b_*^2^/∆*I_b_*^1^~2, were most likely representing the dimers formed through the disulfide bonding of the partially unfolded βLGa protein in 5 M urea. 

As a control measurement, the current blockage events were also recorded at a lower voltage, Ψ= 86 mV (Figure 2H). The peak values of the ∆*I_b_* histogram for the cluster-3 events show that ∆I_b_ = 137 ± 17 pA at Ψ= 86 mV, which is smaller compared to ∆I_b_ = 171 ± 31 pA at 120 mV (Figure 2H, right panel), which was consistent with Equation (1). No big difference was observed for the t_d_ recorded at the two voltages (Figure 2H, bottom panel). The small changes in ∆*I_b_* for the cluster-1 events as well as the time, *t_d_*, for the two voltages were consistent with the results reported in our earlier studies [39]; that cluster-1 was most likely representing events from the partially unfolded βLGa monomer translocation through a nanopore, together with the events of collision and noise spikes.

#### 3.1.2. ßLGa Incubated on Day 29

**Figure 3:**
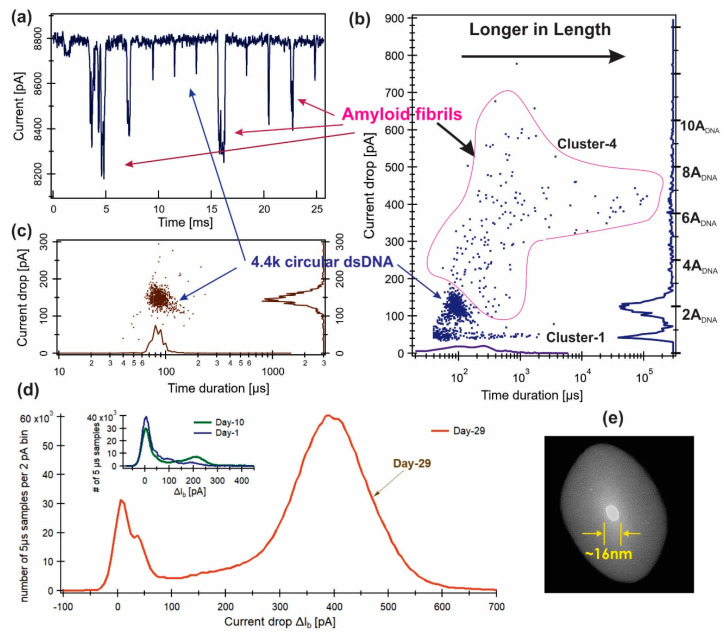
Nanopore measurements for the “mature” amyloid fibrils (Day 29 of incubation) at conditions 1.6 M KCl, 20% Glycerol, 10 mM Tris-EDTA buffer at pH.7. (**a**) Typical events, (**b**) event distribution, (**c**) dsDNA control, (**d**) instantaneous time distribution of blockade current, Δ*I*_b_, over all events selected (including 200 samples or 1 ms before and after each event). The same plot for the Day-1 and Day-10 samples from Figure 2 are shown in the insert. (**e**) The ~16 nm diameter pore nanopore used for the experiment. All measurements were performed at Ψ = 120 mV.

A larger nanopore (~16 nm diameter, Figure 3e) was used for this experiment. A circular relaxed 4.4 kbp dsDNA, as a reference molecule, was measured first. The cis chamber solution was washed (not completely; some DNA molecules were still present) then the ßLGa Day-29 sample was added. 

For the Day-29 sample, events of very large ∆*I*_b_ (>200 pA) and long *t*_d_ (~ms) were observed (Figure 3a) and the distribution of the events is shown in Figure 3b. For the circular dsDNA, the event distribution plot (Figure 3c) shows that the most probable peak values were at ∆*I*_b_ = 145 ± 10 pA and *t*_d_ = 84 ± 4 µs. The ∆*I*_b_ histogram for the Day-29 sample (Figure 3b) had a major peak at ∆I_b_ ~122±11 pA. This was most likely a peak of mixed 4.4 kbp circular dsDNA together with some events from the βLGa Day-29 sample that could not be resolved. The Day-29 cluster-1 events (Figure 3b) had a current blockage peak value of ∆*I*_b,1_ ≈ 42 pA and t_d,1_ ≈ 50 µs, which was similar to the cluster-1 events observed for the Day-10 and Day-1 samples. The Day-29 cluster-4 for the large current drop events (Figure 3b) had a very broad distribution of ∆*I*_b_, centered at ∆*I*_b,4_ ≈ 400 ± 100 pA. The Day-29 *t*_d,4_ histogram at the bottom axis shows multiple time scales spanning from 200 µs–2 ms (see Figure S1 in S-I for details). The plot of the Day-29 instantaneous time distribution of the blockage current, ∆*I*_b_, over all the selected events (Figure 3d) shows more clearly a large current drop peak at ∆*I*_b,4_ ≈ 400 ± 100 pA. The same plots for the Day-10 and Day-1 data from Figure 2 are in the insert to show the major peak values. 

The open pore current (*I*_0_) for a given nanopore was consistent across all the DNA and βLGa measurements, suggesting that the nanopore area (*A*_po_) remained constant. Using the known cross section area of a dsDNA, $A_{dann}=(1.8\;{nm}^{2})$ [80], the arft hethe nanopore estimated was $A_{po}=2dann/\left(\frac{∆I_{b}}{I_{0}}\right)=236.2{\;nm}^{2}$ (Table A1), with a diameter of 17.8 nm. Using the open pore current, $I_{0}=8.80\;nA,$ the estimated effective thickness of the pore was $H_{eff}=\frac{\Psi \sigma A_{po}}{I_{0}}=32.8\;nm$, therefore the effective volume of the pore was${\;V}_{po}(2MKCl)=A_{po}H_{eff}=7750{\;nm}^{3}$. Interpreting that the cluster-4 events of the Day-29 sample arose from the βLGa filaments, and using the DNA calibrated nanopore geometry of ${\gamma \Lambda }_{Pr}≃\left(\frac{∆I_{b}}{I_{0}}\right)V_{po}$ with $\gamma =1\;for\;long\;filaments\;of\;length>H_{eff}$, the estimated most probable excluded volume of the βLGa fibril in the pore with $H_{eff}=32.8\;nm$ was $\Lambda_{Pr}\left(cluster\;4\right)=352.0\;{nm}^{3}$, which gives an area of 10.7 nm^2^ (Table 1). For the cluster-1 events, $\gamma \Lambda_{Pr}\left(cluster\;1\right)=37.0{\;nm}^{3}$.

#### 3.1.3. AFM and DLS Measurements

**Figure 4:**
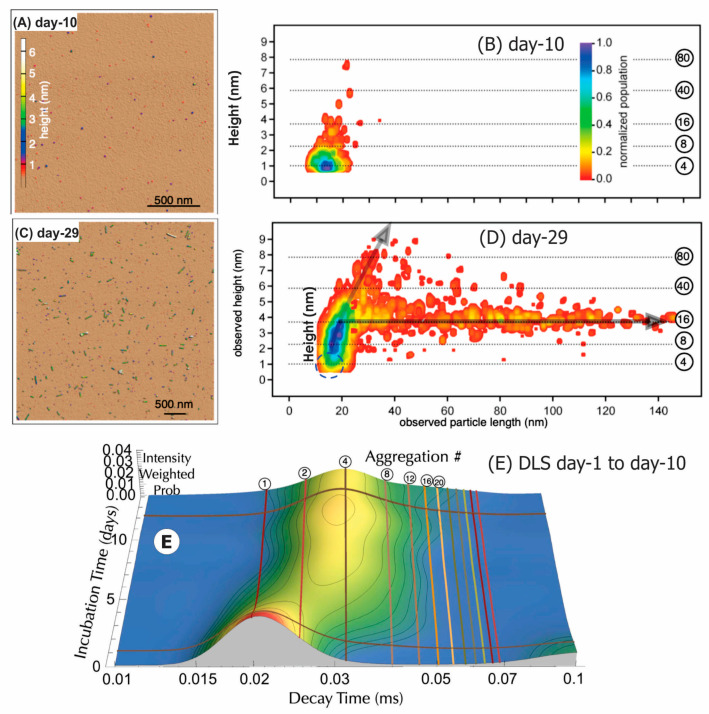
AFM images and histograms of Day-10 and Day-29 samples. For the Day-10 sample (**A**,**B**), the most probable values were *l*_1_ = 11.4 ± 5 nm long, *d*_1_ = 1.5 ± 0.2 nm in diameter were measured. For the Day-29 sample (**C**,**D**), the most probable values were *l*_1_ = 15 ± 5 nm long, *d*_1_ = 1.5 nm in diameter. Second broad peak of fibrils: *l*_4_ = 80 ± 10 nm long, *d*_4_ = 4 ± 0.2 nm in diameter. (**E**) Dynamic light scattering data taken as a function of incubation time from Day 1 to Day 10 under aggregation-prone amyloidogenic conditions. Aggregation number equivalents to the diffusion times are marked by labeled mesh lines.

*AFM measurement of ßLGa Day-10 sample*. The AFM images of the Day-10 βLGa sample on functionalized mica (Figure 4A) and their histograms (Figure 4B) show that the most probable values were length, $l_{3}$ = 11.4 ± 5 nm, and diameter, *d*_3_ = 1.5 ± 0.2 nm. The calculated cross-section area, A^3^_AFM,_ (Day-10) = 1.77 nm^2^ and the estimated volume was V^3^_AFM_(Day-10) ≈ $l_{3}$ × A^3^_AFM_ ≈ 20 nm^3^. An aggregated species with a larger diameter (height d = 4.0 ± 0.2 nm) and longer in length, l = 20–40 nm long, was also detected. 

*DLS measurement of Day-1 to Day-10 sample*. Dynamic light scattering (DLS) measurements (Figure 4E) show that most of the βLGa was monomeric on Day 1 and began to form pre-amyloidogenic aggregates around Day 10 of incubation. The most likely aggregated size was tetrameric (aggregation number n = 4), with larger aggregates beginning to form. These observations were consistent with the results by Giurleo et al. [11]; that the Day-10 sample was likely a mixture of partially denatured monomer and aggregation A (tetramers) and started to form aggregation B (octamers). More details about the DLS measurement are described in S-II. 

*AFM measurement of the Day-29 sample*. The Day-29 βLGa sample on the mica surface (Figure 4C) and the histograms (Figure 4D) show a main peak of length *l*_3_ = 15 ± 5 nm and *d*_3_ = 1.5 nm in diameter. The aggregated species for the broad peak had values of *l*_4_ = 80 ± 10 nm and *d*_4_ = 4 ± 0.2 nm. For comparison with nanopore measurements, the calculated cross-sectional areas and their volumes are listed in Table 1.

#### 3.1.4. Summary of βLGa Aggregation Characterization under Partially Denaturing Condition

*The Day-1 and Day-10 sample*. The above results and data analysis suggested (1) the cluster-1 events, ∆*I*_b,1_ ≈ 45 pA, were partially denatured βLGa monomers; (2) the cluster-2 events measured for the Day-1 sample with ∆*I*_b,2_ (Day1) ~100 pA were likely the βLGa dimers; (3) the cluster-3 events (Figure 2C) with ∆*I*_b,3_ ≈ 150–200 pA were most likely from tetramers, and possibly some trimers. 

*The Day-29 sample*. The cluster-4 events from the Day-29 sample (Figure 3) with peak values of ∆*I*_b_ ≈ 400 ± 100 pA and t_d_ ~200 µs to 2 ms were most likely produced by large βLGa fibrils. Using the most probable length of the fibril measured by AFM, *l*_4_ = 80 $>H_{eff}(32.8\;nm)$, the cross-section area of the fibril can be estimated by A_βLGa_ ^4^$\;\approx \left(\frac{∆I_{b}}{I_{0}}\right)A_{po}=0.0455\times 236.2\;{nm}^{2}=10.7{\;nm}^{2}$ (or diameter = 3.7 nm). This was very close to the AFM-measured diameter *d*_4_ = 4.0 ± 0.2 nm (area = 12.6 nm^2^, see Table 1). We conclude here that the cluster-4 events represent amyloid protofibrils/filaments, a species of amyloid with a diameter of 3–6 nm and length <100 nm. Note that, here, we used the cross-section area of a dsDNA: *A*_DNA_ = (1.8 nm^2^) [80]. If we used a diameter for a dsDNA obtained from X-ray diffraction, 2.0 nm [81] and *A*_DNA_=3.14 nm^2^, the estimated diameter of the pore would be unrealistically large, 23.1 nm, not consistent with the TEM image and the observed open pore current value.

### 3.2. Nanopore Characterization of βLGa Oligomerization at pH 4.6

Next, we show that a solid-state nanopore device can be used to characterize the βLGa native-state oligomerization at pH 4.6 and different temperatures (Figure 1A). Early studies have shown that below room temperature, in the pH range of 3.7 to 5.2, βLGa reversibly forms larger oligomers [12,82]. This self-association process is at its maximum around pH 4.6, just below the isoelectric point. The static light scattering data indicated that the large oligomers cooperatively formed octamers, and the radius of gyration, deduced from small-angle X-ray scattering (SAXS), indicated a compact cubic arrangement of eight monomers [12,82]. The BLG protein self-association has also been studied as a function of the solution temperature (1 °C–27 °C) with no salt present at pH 4.7 by proton magnetic dispersion (MRD) [82], and the results have shown that the BLG are in dimer–octamer equilibrium. Combining static and DLS [12] at pH 4.3, in the temperature range 8 °C to 35 °C, βLGa aggregation has been measured as a function of the salt concentration. These studies have shown that the βLGa would be in a stable dimer form at 2 M KCl. However, at low salt 0.1–0.5 M conditions, the βLGa would aggregate to tetramers, hexamers, and octamers at the same temperature, with the average aggregation number increasing with a lower temperature.

**Figure 5:**
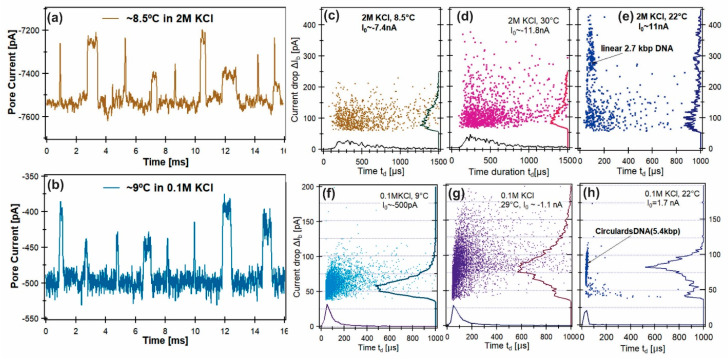
Examples of current blockage events recorded for βLGa at pH 4.6 and ~8.5 °C in 2 M KCl (**a**) and 9 °C in 0.1 M KCl (**b**). Event distribution plots, Δ*I*_b_ vs. *t_d_* in 2 M KCl for BLGa at ~8.5 °C (**c**), at ~30 °C (**d**), and (**e**) for linear 2.7 kbp dsDNA at ~22 °C I; in 0.1 M KCl for BLGa at ~9 °C (**f**), at ~29 °C (**g**), and for circular 5.4 kbp dsDNA at ~22 °C (**h**). The histograms of Δ*I*_b_ are shown on the right axes and *t_d_* are on the bottom axes. Protein concentration in the cis chamber was ~200 nM at pH 4.6. More scattered plots and examples of events can be found in S-I (Figures S3 and S4).

**Figure 6:**
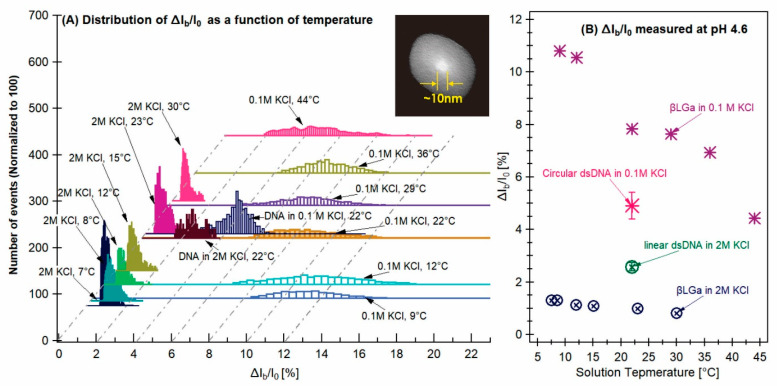
(**A**) The event distributions (normalized to 100) of Δ*I_b_*/*I*_0_ for the entire set of experiments performed. The TEM image of the ~10 nm nanopore used for the set of experiments is shown in the insert. (**B**) Peak values of Δ*I_b_*/*I*_0_ as a function of temperature for all the *β*LGa sample measured at pH 4.6 together with the dsDNA [2 M KCl (⊗) and 0.1 M KCl (*)] with the same nanopore.

The nanopore measurements of the βLGa oligomerization (Figure 5 and Figure 6) were performed as a function of the solution temperature under two salt conditions, 2 M and 0.1 M KCl, both with 100 mM pH.4.6 acetate buffer. A 2.7 kbp linear dsDNA (1*A*_DNA_) in 2 M KCl and a circular 5.4 kbp dsDNA (2*A*_DNA_) in 0.1 M KCl solution were used as reference molecules. The βLGa concentration was ~200 nM in the cis chamber. The solution temperature was varied from 5 °C to 45 °C. A single ~10 nm pore (Figure 6A insert) was used for all the measurements presented in Figure 5 and Figure 6. The open pore current was the same at the end of the experiment under the same solution conditions; therefore, the geometry of the nanopore remained the same for all the data presented in Figure 5 and Figure 6.

At pH 4.6, βLGa protein is positively charged, thus the *trans* chamber bias was switched to negative, and the open pore currents were negative (Figure 5a,b). For the negatively charged dsDNA molecules, the trans chamber was positively biased. The examples of the event distributions for βLGa in 2 M KCl at 30 °C (Figure 5c) and at 8.5 °C (Figure 5d) show that the average blockade current, Δ*I*_b_ (on the right axis), had similar distributions. The peak values were Δ*I*_b_ = 64 ± 11 pA at 8.5 °C (Figure 5c) and Δ*I*_b_ = 65 ± 15 pA at 30 °C (Figure 5d). In 0.1 M KCl, the event distribution for βLGa at 29 °C (Figure 5f) and at 9 °C (Figure 5g) show that the distribution of Δ*I*_b_ changed significantly. The peak values were Δ*I*_b_ = 62 ± 11 pA at 9 °C (Figure 5f) and 82 ± 15 pA at 30 °C (Figure 5g). The control experiment performed with the linear dsDNA (Figure 5e) at room temperature (~22 °C) in 2 M KCl shows that the most probable current blockage amplitude was Δ*I*_b_~280 pA and *t*_d_ = 87 ± 17 µs. In 0.1 M KCl, the circular dsDNA in Figure 5h shows a very narrow peak, with values of Δ*I*_b_ = 83 ± 8 pA (*100.6 pA after amplitude correction [23]) and *t_d_* = 31 ± 8 µs. 

The open pore current, *I*_0_, for both 2 M and 0.1 M KCl solution increased with temperature (Figure S2), consistent with the thermal increase in the solution conductivity. To account for the solution conductivity variation with temperature, we plotted the ratio of the current blockage values to their open pore current, Δ*I*_b_/*I*_0_, in Figure 6. For the βLGa protein in 2 M salt solution at pH 4.6, the distributions of Δ*I*_b_/*I*_0_ did not change significantly as temperature varied from 7 to 30 °C, showing narrow peaks around Δ*I*_b_/*I*_0_ = 0.7%. This was consistent with an earlier report that the βLGa would be in a stable dimer form at 2 M KCl [12]. For βLGa in 0.1 M KCl, the distributions of Δ*I*_b_/*I*_0_ in Figure 6A show the values of Δ*I*_b_/*I*_0_ ranged from 2% to 14%, indicating that in 0.1 M KCl, the βLGa protein molecules could be in self-association or in oligomerization forms. 

All the peak values of Δ*I*_b_/*I*_0_ for the entire set of βLGa samples together with the dsDNA performed with the same nanopore are shown in Figure 6B. For the βLGa in 2 M KCl, the most probable value of Δ*I*_b_/*I*_0_ was between 0.5–0.7% as the solution temperature was decreased from 30 °C to 8 °C (⊗ in Figure 6B bottom). This measurement indicates that the majority of the βLGa protein molecules are likely in a stable monomer or dimer form in the temperature range tested. In 0.1 M KCl, the ratio of the Δ*I*_b_/*I*_0_ values increased from 4.5% at 44 °C to 11% at 9 °C, suggesting the aggregation number became larger as the temperature was lowered. This was consistent with the earlier results measured using the MRD and DLS methods [12,82]. 

Below, we offer an explanation as to why a low salt concentration favors βLGa oligomerization and why the aggregation number became larger as the temperature was lowered. βLGa has positively and negatively charged regions, giving rise to a dipole moment that has been experimentally measured to be ~700 Debye [83]. The contribution of the dipole moment to the βLGa–βLGa interactions should increase at low salt concentrations and lower temperatures, leading to more oligomerization.

Next, we estimate the excluded volumes, ${\gamma \Lambda }_{Pr}$, from our nanopore measurements. The values of Δ*I*_b_/*I*_0_, measured as ~2.6% for the linear dsDNA in 2 M (⊗) and ~4.9% (*5.9% after correction) for the circular dsDNA in 0.1 M KCl, were consistent with Equation (1). For a dsDNA molecule with a contour length${\;L}_{DNA}\gg H_{eff}$, Equation (1) leads to $∆I_{b}/I_{0}\sim A_{DNA}/A_{pore}$. Using *A*_dsDNA_ = (1.8 nm^2^) [80] and *A*_CirdsDNA_ = 2 × 1.8=3.6 nm^2^ for circular dsDNA, the product of the excluded volume and shape factor: ${\gamma \Lambda }_{Pr}≃\left(\frac{∆I_{b}}{I_{0}}\right)V_{po}$ of βLGa protein in 0.1 M KCl and in 2 M KCl at pH 4.6 at different temperatures can be estimated (Figure S5). In 0.1 M KCl, the ${\gamma \Lambda }_{Pr}=30.5\sim 74.3\;{nm}^{3}$. In 2 M KCl, the ${\gamma \Lambda }_{Pr}=6.6\sim 10.5\;{nm}^{3}.\;$The ratios of ${\gamma \Lambda }_{Pr}$ in 0.1 M to 2 M were about 4 to 8 (low temperature) (Figure S5), this was consistent with the expected aggregation numbers. 

However, the estimated values of ${\gamma \Lambda }_{Pr}=8.6\sim 11\;{nm}^{3}$ in 2 M KCl (Figure S5) were only about half the value of $V_{pr}=$ 22.2 nm^3^ that was calculated by adding the volumes of the amino acids together for a monomer. Furthermore, if we take the dsDNA diameter as 2.2 nm (A_dsDNA_ = 3.8 nm^2^), the estimated values of ${\gamma \Lambda }_{Pr}\sim 25.8\;to\;40.9\;{nm}^{3}$ (Figure S5) are approximately four times larger. This suggests that this study shows a nanopore device can estimate the relative volumes of proteins (aggregation states) for comparison; however, is not capable of measuring the absolute volume of a protein. The major reason for this was that this value, ${\gamma \Lambda }_{Pr},$ was calculated based on the volume of a nanopore, $V_{po}=A_{po}H_{eff},\;$and this effective pore volume was difficult to determine accurately under the experimental conditions. The other reasons include the cross-section area of the reference dsDNA molecule in solution was not precisely known and the area of the dsDNA could have become larger at low pH [84], and there was a possibility that the shape of the βLGa protein molecule could change under the electric field strength in a nanopore under the experimental conditions.

In summary, by measuring Δ*I*_b_/*I*_0_ in the same nanopore together with reference dsDNA molecules, we successfully estimated the native-state oligomerization at pH 4.6 under different solution conditions. Our measurement and analysis showed that in 0.1 MKCl, βLGa molecules were likely in the form of tetramers (*n* = 4 at high temperature), hexamers (*n* = 6), and octamers (*n* = 8 at low temperature), and are likely not homogeneous or single species. This set of experiments suggests that a solid-state nanopore device can be used in future studies of the dissociation of protein aggregation.

However, this study showed that the limitation of a nanopore measurement is to measure the absolute volume of a protein molecule, $\Lambda_{Pr}$, because it could depend on the nanopore geometry and the geometry of the calibration dsDNA molecules. Further experiments showed that the ${\gamma \Lambda }_{Pr}$, estimated by the nanopore method, also depended on the applied voltages [85]. The advantage of using dsDNA and cir-dsDNA in this study was that the events from the calibration molecule were easily distinguished from the analyte molecules. A better calibration would involve known particles with known geometry nanoparticles of spherical and rod shapes; however, such an approach would necessitate completing the calibration in series with the measurement rather than in parallel.

## 4. Conclusions

This study has demonstrated that a silicon nitride nanopore device can be used to characterize ß-lactoglobulin variant A (βLGa) protein amyloid formation in salt solution under partial denaturation conditions at pH 7.0 and native state self-association oligomerization at pH 4.6. Our results showed that by measuring the relative current blockage amplitudes combined with using a dsDNA molecule as a reference, we can estimate the βLGa protein aggregation state, including the aggregation number and dimensions. This work also indicates that a solid-state nanopore device can also be used for future studies of protein aggregation.

*The advantages and weaknesses of using the nanopore method to evaluate protein aggregation*. Comparing other ensemble methods as well as single molecule techniques at the present time, this study showed that the solid-state nanopore method can characterize protein aggregation close to its native salt solution environment on the single molecule level. This work demonstrated that the nanopore technique is fast; one set of measurements usually takes less than 10 min to record about five thousand events; a small amount of sample is needed (~10 µL of 100 nM or ~10 pM protein); and it can measure protein aggregation under all biological related parameters such as temperature, pH, electric field strength (voltage), and salt concentration. In addition, nanopore measurement can also estimate the relative aggregation state and its distribution quantitatively. To be able to evaluate protein aggregation under these conditions will be important and valuable and could improve our understanding of protein aggregation mechanisms and allow for the development of new approaches for the prevention and dissociation of amyloid formation and better diagnostics for protein aggregation-related devastating diseases. The limitation of this technique, based on this study, is that the estimated volume of the protein molecules depended on the calibration molecule, the nanopore geometry, and applied voltage. Further investigation is needed to measure the size and shape of the aggregated proteins precisely. 

## Figures and Tables

**Table 1:** Summary of parameters measured and estimated for the Day-1, Day-10, and Day-29 samples.

	Method	Parameters	Cluster-1	Cluster-2	Cluster-3	Cluster-4
**Day1**	nanopore	Δ$I_{b}$ (pA)	44 ± 6 pA	98 ± 14 pA	158 ± 29 pA	
		$t_{d}$ (µs)	66 ± 28 µs	127 ± 35 µs	105 ± 33 µs	
**Day10**	nanopore	Δ$I_{b}$ (pA)	44 ± 6 pA		171 ± 31 pA	
		$t_{d}$ (µs)	130 ± 65 µs		142 ± 41 µs	
	AFM	Diameter (nm)			1.5 ± 0.2 nm	4.0 ± 0.2 nm
		Length (nm)			11.4 ± 5.0 nm	80 ± 10 nm
		Area (nm^2^)			1.77 nm^2^	12.6 nm^2^
**Day29**	nanopore	Δ$I_{b}$ (pA)	42 pA			400 ± 100 pA
		$t_{d}$ (µs)	~50 µs			200 µs~2 ms
		Area (nm^2^)				10.7 nm^2^
		Volume$\;{\gamma \Lambda }_{Pr}$	$37.0{\;nm}^{3}$			*$352\;{nm}^{3}$
	AFM	Diameter (nm)			1.5 nm	4 ± 0.2 nm
		Length (nm)			15 ± 5 nm	80 ± 10 nm
		area			1.77 nm^2^	12.6 nm^2^
		volume			26.5 nm^3^	1004.8 nm^3^
**dsDNA**	nanopore	Δ$I_{b}$ (pA) *I*_0_ (nA)	145 ± 10 pA 9.51 nA			122 ± 11 pA 8.8 nA
		$t_{d}$ (µs)	84 ± 4 µs			
		Diameter (nm)				1.5 nm
		area				1.8 nm^2^

*$352\;{nm}^{3}\;of$ volume$\;{\gamma \Lambda }_{Pr}$ is the volume of the portion of a long βLGa filament inside the pore.

## Data Availability

Data supporting reported results can be provided upon request.

## Supplementary Materials

The following supporting information can be downloaded at https://www.mdpi.com/article/10.3390/s24010081/s1, Figure S1: The time duration (*t*_d,4_) histogram for the Day-29 βLGa sample; Figure S2. Open pore current, I_0_, at different temperatures in 0.1 M KCl and in 2 M KCl; Figure S3. Plots of $∆I_{b}\;vs.\;t_{d}$ and example of events for βLGa protein in 0.1 M KCl; Figure S4. Plots of $∆I_{b}\;vs.\;t_{d}$ and example of events for βLGa protein in 2 M KCl; Figure S5. The estimated product of the excluded protein volume.

## Appendix A

Solution conductivity used for calculations and experiments. For the solution of 2 M KCl with 5 M urea and 10 mM phosphate buffer at pH 7 and room temperature, conductivity σ~120 mS/cm. For the solution of 1.6 M KCl 20% Glycerol 10mM phosphate buffer at pH7, *σ*~110 mS/cm. At room temperature, 2 M KCl with 100 mM acetate buffer at pH 4.6, σ = 150 mS/cm; 0.1 M KCl with 100 mM acetate buffer at pH 4.6, σ = 18.4 mS/cm.

## Appendix B

**Table A1:** Parameters of the 3 nanopores used for this work.

	TEM Diameter	$\mathrm{Diameter}\;\mathrm{by}\;I_{0}$ or DNA	$\mathrm{Area}\;A_{po}$ $\mathrm{by}\;\mathrm{DNA}\;\mathrm{and}\;I_{0}$	$H_{eff}$ $\mathrm{by}\;I_{0}$ or dsDNA	$V_{po}=A_{po}H_{eff}$
Nanopore 1 for Day 1 and Day-10	10 nm	10 nm	$78.5\;{nm}^{2}$	36.5 nm	$2870\;{nm}^{3}$
Nanopore 2 for Day 29	16 nm	17.8 nm	$236.2\;{nm}^{2}$	32.8 nm	$7750\;{nm}^{3}$
Nanopore 3 for pH 4.6	8 nm	9.5 nm (2 M) 9.7 nm (0.1 M)	$69.9\;{nm}^{2}$ $73.5\;{nm}^{2}$	11.4 nm (2 M) $9.36\;nm\;\left(0.1\;M\right)$	$800\;{nm}^{3}$ $688\;{nm}^{3}$
