# Supplementary material for: Aggregation and Oligomerization Characterization of ß-Lactoglobulin Protein Using a Solid-State Nanopore Sensor
Source: Sensors (Basel). 2023 Dec 22;24(1):81. doi: 10.3390/s24010081 (PMC10781269; doi:10.3390/s24010081)
Supplement: Supplementary file 1 [file sensors-24-00081-s001.zip › sensors-2734609-supplementary.pdf]

Supplementary Materials

Title: Aggregation and Oligomerization Characterization of ß-Lactoglobulin Protein Using a Solid-State Nanopore Sensor

Mitu C. Acharjee [1], Brad Ledden [1], Brian Thomas [2], Xianglan He [3], Troy Messina [3,4], Jason Giurleo [3,5], David Talaga [3,6] and Jiali Li [1,2,*]

1 Material Science and Engineering, University of Arkansas at Fayetteville, Fayetteville, AR 72701, USA; mcacharj@uark.edu (M.C.A.); bledden@uark.edu (B.L.)

2 Department of Physics, University of Arkansas, Fayetteville, AR 72701, USA; brt01@uark.edu

3 Department of Chemistry and Chemical Biology, Rutgers, The State University of New Jersey, Piscataway, NJ 08854, USA; xianglhe@eden.rutgers.edu (X.H.); tmessina@rci.rutgers.edu (T.M.); jason.giurleo@regeneron.com (J.G.); talagad@montclair.edu (D.T.)

4 Department of Physics, Berea College, Berea, KY 40404, USA

5 Regeneron Pharmaceuticals, Basking Ridge, NJ 07920, USA

6 Department of Chemistry, Sokol Institute, Montclair State University, Montclair, NJ 07043, USA

* Correspondence: jialili@uark.edu

S-I. The time duration t_d^4(Day29) histogram for the Day-29 βLGa sample (at the bottom axis of Fig. 4c) shows multiple time scales span from t_d^4 ~ 100 μs to 2 ms.

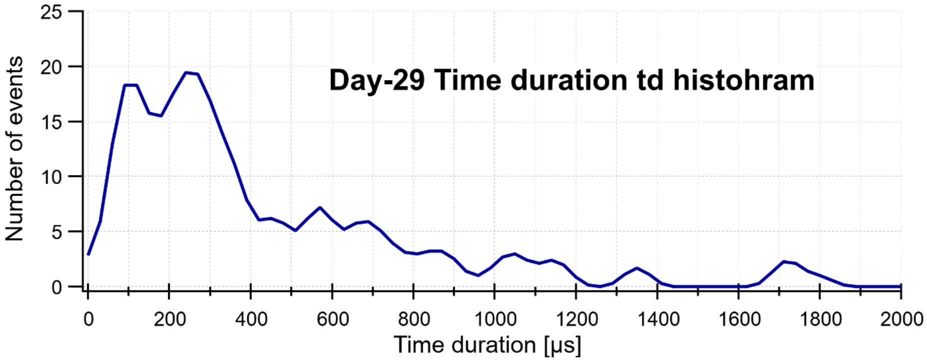

Figure S1. The time duration t_d^4(Day29) histogram for the Day-29 βLGa sample.

S-IIa. ***Details of chemical surface modification to improve the adhesion of protein aggregates to the mica surface for AFM Measurement***. To modify the mica surface, 30 µL of 0.1% (v/v) APTES (3-aminopropyl)triethoxysilane Acros catalog number: 151081000) was applied evenly on freshly cleaved 9.9 mm diameter muscovite mica disk (Ted Pella product number: 50) and allowed to react for 10 minutes. Unreacted APTES was rinsed away with 15 mL Millipore water. The surface was dried with HPLC grade nitrogen gas. The incubated sample was applied evenly on this freshly prepared surface and allowed to adsorb for 10minutes. Unbound species were rinsed away with Millipore water. Residual water was blownaway with nitrogen gas.

S-IIb. ***Details of the DLS measurement***. Fluctuations of scattered light intensity were measured using a homodyne technique. At a particular incubation time x, the intensity correlation function g2(t,x) was measured by a modified Nicomp Model 380 Particle Size Analyzer (Particle Sizing Systems). Scattered light from the incident laser (λ=532 nm) was collected orthogonally (θ=90°). Round borosilicate glass cuvettes (Kimble Glass) were used for all DLS measurements. For the DLS study, 250 µL of incubated sample was placed in a clean dry cuvette. Twenty correlation functions were measured sequentially for 30 s apiece for the incubated sample. The cuvette chamber was held at a constant temperature of 37 °C.

DSL data were globally fitted onto a 50-point grid with logarithmically spaced decay times Γ^{-1} ranging from 0.001 to 65 ms. A particle's decay time is related to the translational diffusion constant through the scattering vector q, such that $D=\Gamma/q^2$, where $q=4\pi/\lambda \sin(\theta/2)$. The diffusion constant can then be converted into Stokes hydrodynamic radius (R_H) using the Stokes–Einstein relation $R_H=(k_BT)/(6\pi\eta_0 D)$, assuming a spherical shape. For these experiments, T is the temperature, η_0 is the refractive index of the buffer, and k_B is the Boltzmann constant. The characteristic density of the partially unfolded monomer determined from assignment of a urea titration DLS experiment at 5.0 M was used to scale the oligomer sizes.

S-III. Open pore currents $_0$ as a function of temperature in 0.1M KCl and 2M solutions at pH 4.6.

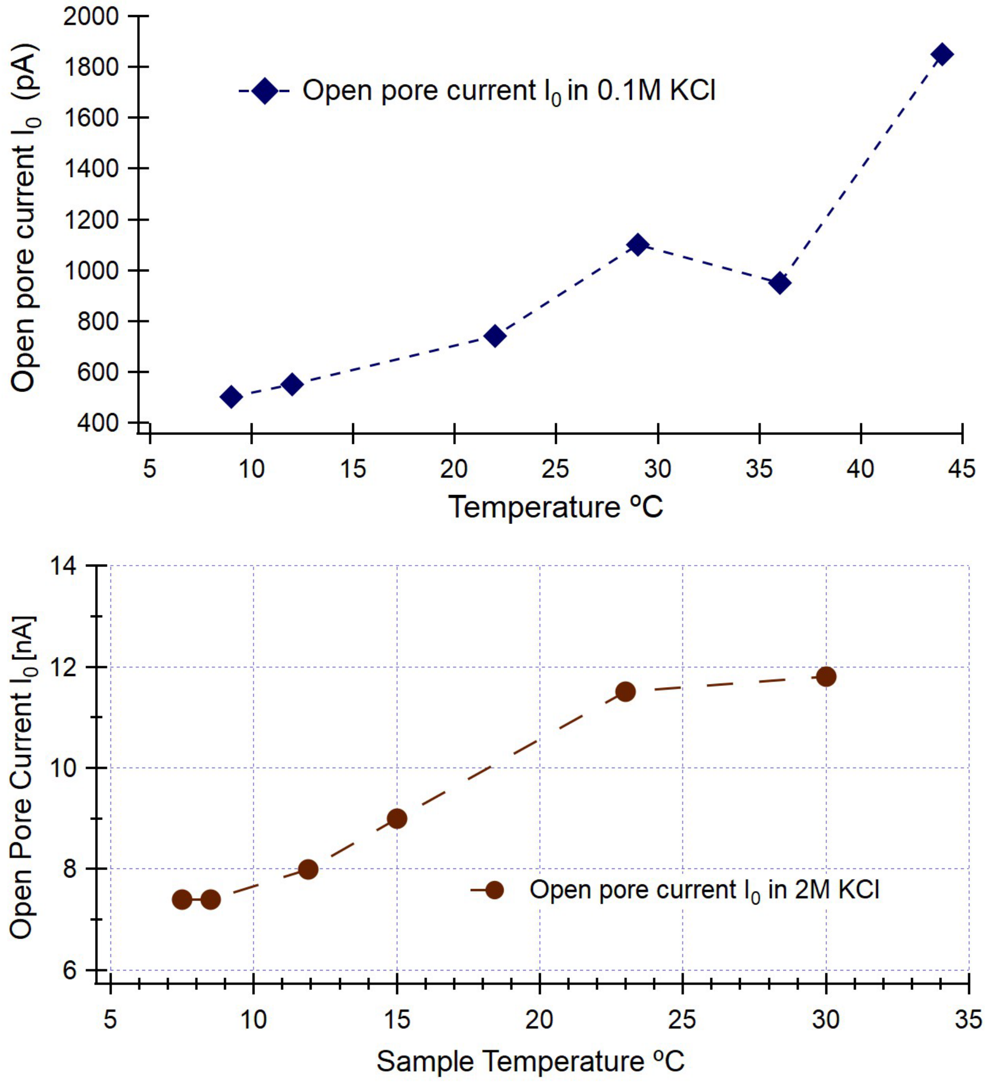

Figure S2. Open pore current I_0 at different temperatures in 0.1M KCl (A) and in 2M KCl (B)

S-IV. Scattered plots, ΔI_b t_d for βLGa protein in 2M KCl and 0.1M KCl at pH 4.6.

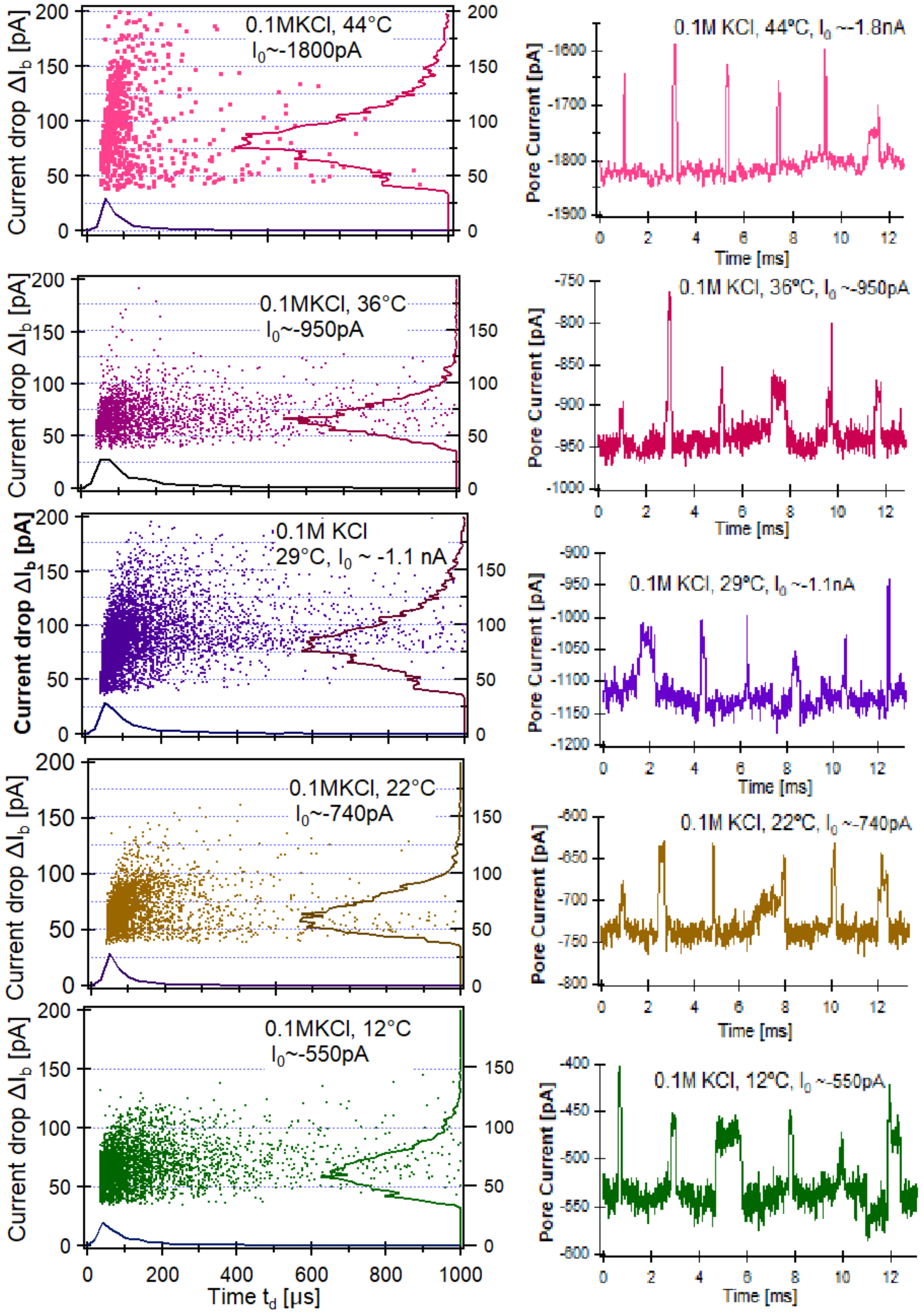

Figure S3. Scattered plots, ΔI_b t_d and example of events for βLGa protein in 0.1M KCl.

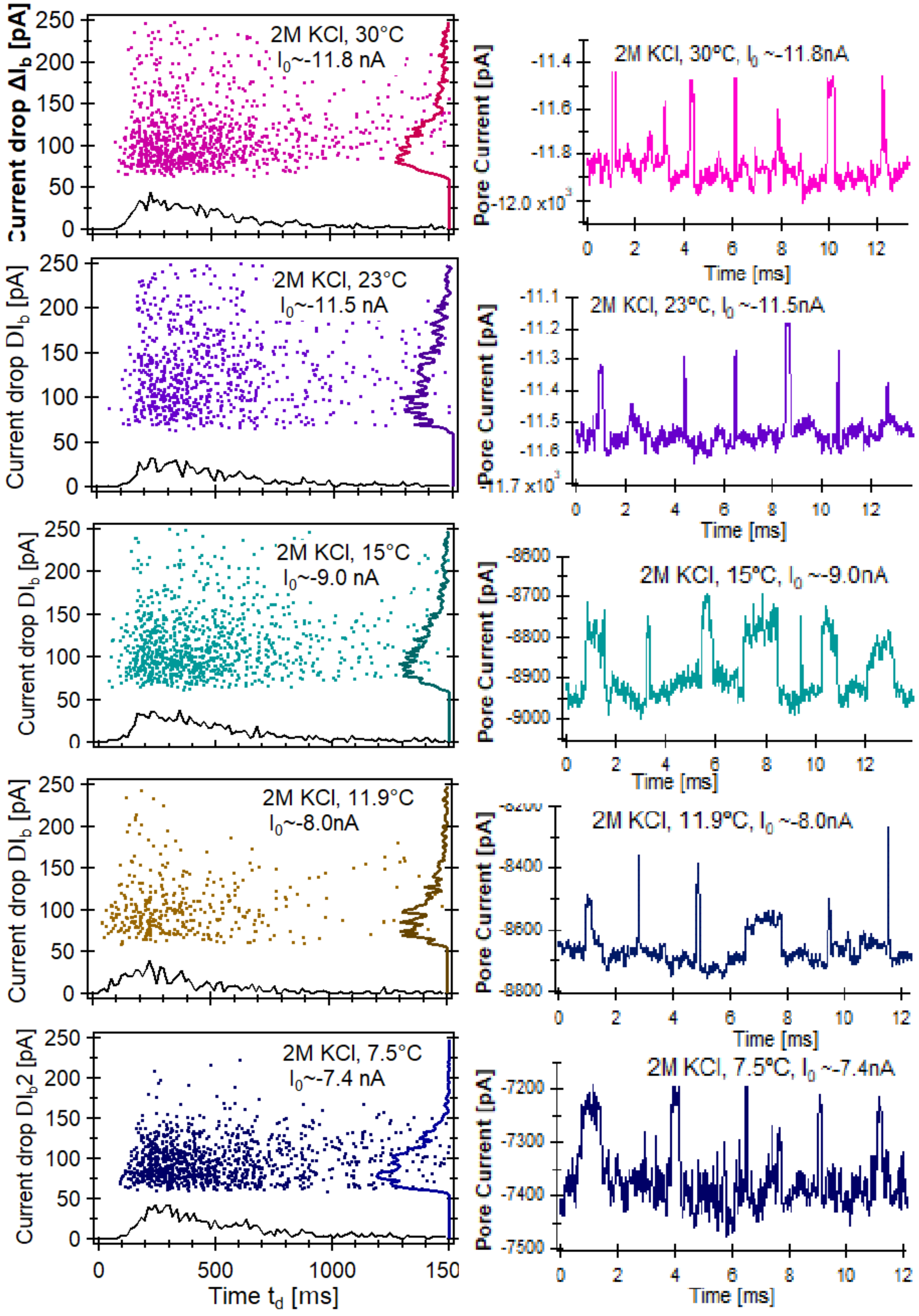

Figure S4. Scattered plots, ΔI_b t_d and example of events for βLGa protein in 2M KCl

S-V. The estimated product of the excluded protein volume and shape factor $\gamma\Lambda$ of βLGa protein in 0.1M KCl and in 2M KCl at pH 4.6 at different temperatures.

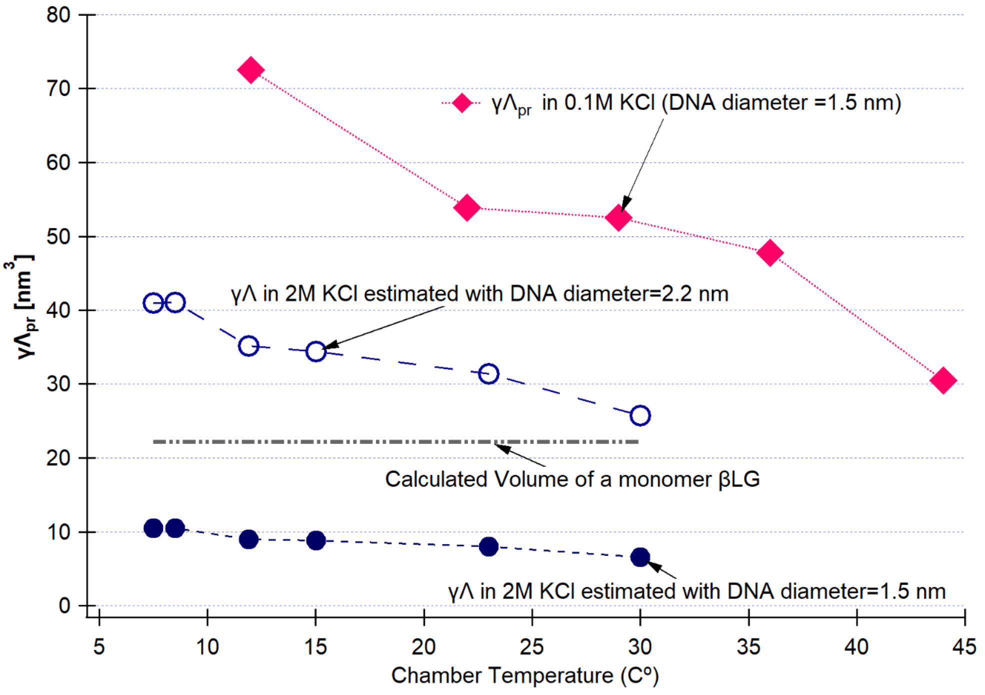

Figure S5. The estimated product of the excluded protein volume and shape factor: $\gamma\Lambda_{Pr} \simeq \left(\frac{\Delta I_b}{I_0}\right) V_{po}$, of βLGa protein in 0.1M KCl () and in 2M KCl at pH 4.6 at different temperatures. Calculation was based on the $\frac{\Delta I_b}{I_0}$ data presented in Fig. 6B. The nanopore volumes used were $V_{po} = 799.7$ ^{3} in 2M KCl and 688.0 ^{3}in 0.1M KCl (Table 2). The calculated volume of a monomer V_{pr} =22.2 nm^3 was calculated by adding the volume of amino acids together for a βLGa monomer.
